# The effect of child malnourishment on measles spread amidst the COVID-19 pandemic in Afghanistan

**DOI:** 10.1016/j.amsu.2022.103798

**Published:** 2022-05-18

**Authors:** Yumna Salman, Sean Kaisser Shaeen, Hira Anas Khan, Zarmina Islam, Mohammad Yasir Essar

**Affiliations:** Faculty of Medicine, Dow Medical College, Dow University of Health Sciences, Pakistan; Kabul University of Medical Sciences, Kabul, Afghanistan

**Keywords:** Afghanistan, Children malnourishment, COVID-19, Measles, Co-infections, Humanitarian crisis

## Abstract

Child malnourishment is a long-lasting concern that Afghanistan has been facing for many years now. This major factor amongst countless others like growing socioeconomic disparity, ineffective healthcare due to lack of funding and political instability has caused increase in nutritional instability through Afghanistan. This has increased the likelihood of numerous malnourished children contracting deadly infectious diseases like measles. Despite receiving nutritional aid, vaccines (reduce measles spread) and funding from international organizations much of these efforts have fell short due to the political instability and lack of sustained support. Emergence of COVID-19 has further intensified the already existing challenges faced by vulnerable Afghan children. The pandemic has impeded with the eradication of measles and vaccine coverage as much of the funding and attention has shifted to containing COVID-19 spread posing a greater threat for malnourished children. The combined effect of both infections has exacerbated and increased mortality in malnourished children as rate of measle spread increases. Afghan healthcare systems are now struggling more as much of their efforts are ineffectual due to lack of facilities and resources.

## 1 Introduction

Measles, a single stranded RNA virus resides from the Morbillivirus genus; a subtype of Paramyxovirus family [1] has resulted in over 140,000 deaths in 2018 alone, mostly affecting malnourished children under the age of five [2]. The highly contagious virus transmits through direct contact and is airborne^2 .^ During the prodromal period, it presents with malaise and fever accompanied with conjunctivitis, cough and coryza. Subsequently, clinical signs appear 10–14 days after being infected with measles [3] and kolpik's spots and maculopapular rash can arise on the proceeding days [1]. Death occurs mainly due to severe complications like blindness, encephalitis, and pneumonia [2]. Measle infections which mostly affects children also commonly occurs in malnourished children, especially those with vitamin A deficiency or weakened immune system because of human immunodeficiency virus or acquired immunodeficiency syndrome [2]. Both factors mentioned make it difficult for malnourished children to fight post-measles complications like pneumonia [4].

Afghanistan is facing a humanitarian crisis, whereby malnourishment in children, and a rise in measles cases have exacerbated healthcare concerns amidst COVID-19. On January 1st, 2021, to January 29th, 2022, out of 35,319 suspected measles cases, 3221 cases (9%) were confirmed via IgM-ELISA in Afghanistan [5]. Additionally, in Helman and Herat, two hospitals report that 800 children have severe acute malnutrition from the period of January to February 2022 [4]. Afghan children are vulnerable to infectious diseases due to unprecedented food insecurity, and lack of appropriate healthcare infrastructure. Worldwide measles surveillance has declined over the past two decades. More than 22 million children were reported to have not received their first measles vaccine in 2020, which is 3 million more than in 2019 [6]. The principal cause for this is after the emergence of the pandemic, surveillance and funding efforts were shifted to delaying the spread of COVID-19; this has caused a global decrease in measle vaccine coverage. In Afghanistan, only 353 measle cases were reported in 2019 [7] (prior to the pandemic) however with the emergence of the pandemic, 35,319 suspected cases were reported alone in January 2021 [5]. The escalation of measle related cases indicates that international efforts are prioritising COVID-19 surveillance, as in the same year, 1.4 million doses of Johnson & Johnson COVID-19 vaccines were donated by Unites States to the Afghan government, 2021 [8]. Thusly, measles-related deaths have increased following the pandemic in various countries, like Afghanistan (mainly malnourished Afghan children) due to decrease in measle surveillance and vaccine funding.

Prolonged civil unrest and sanctions imposed by global leaders have contributed significantly to the progressively deteriorating situation in Afghanistan, as seen through the rapid spread of measles in least 7 out of 30 of the most war-afflicted provinces of the Afghanistan [9]. Currently, 90% of Afghanistan's population to live on less than $2 per day [10]. With a drastic drop in daily labour wages, due to lack of work, and inflation in food prices of rice and sugar (increased by 7–12%) [11]. Many parents are unable to meet basic daily needs or provide food for there already malnourished children [11]. The resulting nutritional deficit makes such children more susceptible to contracting measles than children with comparatively better resources. Concurrently, increased evacuation efforts have intensified the spread of COVID-19 and measles among Afghan evacuees posing a potential global threat [3].

Thus, this article aims to comment on the effect of child malnourishment and its contribution to measle spread, amidst COVID-19 in Afghanistan along with recommendations to curb this spread.

## 2 Historical progress

Historically, Afghanistan has been battling measles outbreaks for years, across a span of nine years (1354-1362)1,39,436 children were admitted to Indria Gandhi hospital of Child Health (IGICH), Kabul, where 46.8% of these children were suffering from preventable diseases like tuberculosis and measles, of these, 74% were malnourished children under the age of five [12]. Hence, a strong correlation has been drawn between malnourished children and spread infectious diseases like measles amongst them.

In 2002, acute and chronic malnourishment was widespread amongst the Afghan children with an anticipation of approximately 2 million refugees to return to Afghanistan from neighbouring countries [13]. Hence, multiple international organizations (World Health Organisation and [WHO] and UNICEF) expected a mass measles outbreak. Concerning this matter, WHO and UNICEF increased $8 million to support measle vaccination campaign and targeted vaccination of children aged 6 month- 12 years [13]. Nationwide vaccination campaigns were executed, where high-risk districts with the largest number of malnourished children and those in isolated and obscure areas were vaccinated first. To ensure this containment, each district reviewed there measles vaccination coverage in October 2002; it was recorded that all children were vaccinated with a mean coverage of <80% [13].

## 3 Challenges and implications

Afghanistan's measles crisis seems far from eradication due to numerous issues such as the country's increasing malnourishment rates and its effects on children's health, insufficient data and growing socioeconomic disparity. A weak agriculture industry with no compensation through global funding has led to an estimated 3.2 million children under the age of 5 suffering from acute malnourishment, according to UNICEF [2]. The resulting nutritional deficit makes such children 24 times more susceptible to contracting measles than normal children [14].

The long-standing issue of child malnourishment has alarmingly escalated during the COVID-19 pandemic. Protein energy malnutrition is, in fact, a primary cause of immune deficiency worldwide [15] which aids the progression of both COVID-19 and measles.

Wasting and stunting in malnourished children has also been associated with increased child mortality due to infectious diseases, such as pneumonia [15]. Bearing in mind COVID-19's long-lasting detrimental effects on overall health, especially lung function [16]and pneumonia as a complication of measles being an independent statistically significant risk factor for death [17], this situation gives a much bleaker outlook to the current measles outbreak with millions of children at risk of life-threatening complications. Unsurprisingly, case fatality rates have risen to 8–13%, as estimated by WHO [2].

The subsequent rise in measles cases is also attributed to the need to divert the already scarce funds to COVID-19 management due to the inability of the economy to support both COVID-19 and measles programs. With Afghanistan's COVID-19 case count reaching 177,663 on March 30th, 2022, COVID-19 vaccination programs have been prioritized, with 5,751,015 doses administered till March 21st, 2022 [18]. The discontinuation of mass measles vaccination programs to prevent COVID-19 transmission has led to low vaccination coverage of a mere 66% for MCV1 and 43% for MCV2 in 2020, instead of the recommended 95% [2]. Following the Taliban takeover in August 2021, WHO has supported a measles immunization campaign for 1.5 million children in December 2021 [19]. While this is a step in the right direction, a consistent program has not been put in place and Afghanistan still looks towards WHO and other sources for help. The lack of immunization among children further increases the risk for measles transmission, especially in malnourished children.

Prevention of measles is particularly challenging due to inadequate resources for sufficient epidemiological studies. The understatement of cases and mortality due to low testing rates, particularly in rural areas, make contact tracing and local management according to major causative factors specific to those areas difficult.

The pandemic has also amplified socioeconomic disparity between populations in rural and urban settlements. Large families with inadequate housing, poor hygiene, low vaccine coverage, and inaccessibility to adequate healthcare are prime targets of the virus’ high transmissibility. Although double dose vitamin-A supplementation has been reported to decrease child mortality in measles patients by 62% [20], malnourishment, and poverty in rural areas rid people of such advantage. Non-compliance with safety protocols and a lack of awareness contribute to a dangerous combination of rising COVID-19 and measles cases.

## 4 Efforts and recommendations

There are various efforts being simultaneously conducted by the current government and different international humanitarian organizations to combat the current measles outbreak taking place in Afghanistan. To start, the Taliban government joined a high-level meeting held in Doha, Qatar with representatives from its host country, WHO, UNICEF, and other organizations to discuss possible solutions to the current crises as well as ways to ensure health care for as many Afghan citizens as possible without discrimination [21]. The WHO itself has launched a measles vaccination campaign to directly combat the rising number of measles cases by providing and vaccinating more than 1.2 million children under the age of five [22]. Additionally, the WHO is supporting the Ministry of Public Health and various provincial health authorities by providing technical advice, training for healthcare workers with corresponding funds as well as providing resources for the cost of the operation i.e., supplies and logistics for the vaccination campaign [23]. Lastly, given the current humanitarian crises in Afghanistan and the lack of nutrition, organizations have provided vitamin A supplementation for children aged 6–59 months old as part of an emergency response to treat more than 1000 hospitalized children suffering from severe acute malnutrition [5]. An additional 34.6 tons of life saving health supplies was also airlifted to treat roughly 15,000 patients for three months [5]. This is essential to combat measles as malnutrition, specifically vitamin A deficiency, is a risk factor for children of similar age groups [14,24].

Measles in Afghanistan is a multivariate problem and thus requires multiple solutions from different angles to address it. First and foremost, is Afghanistan's struggling health care system needs to be revitalized. This can be done by training more locals to bolster the heavily understaffed facilities that are still functioning as well as by procuring funds to train said staff. It is also essential to fund the already existing staff as many of them are underpaid or simply not paid at all, this along with the dangers of the environment has caused many healthcare workers to avoid working all together. Although there have been many drives to gather donations such as when the United Nations humanitarian appeal raised 1.85 billion euros, it still falls significantly lower than the 3.3 billion euros that was the initial goal [25]. One of the reasons why the drive fell short is because of international sanctions being made on the Taliban government as the World Bank suspended more than 600 million euros in aid projects due to the same governments sudden decision to renegade on its earlier promise to let female students return to secondary school [25]. Therefore, it is imperative for the sake of the Afghan citizens that international leaders work together with the Taliban government in order to differentiate and separate financial aid for healthcare and politics. This should be done while also educating the government in the need for vaccinations to dispel any myths surrounding the measles vaccine which can discourage citizens from becoming vaccinated.

Lastly food and vitamin A supplementation should go hand in hand with the measles vaccination as both contribute significantly in reducing the rise of measles cases and would have a great impact as Vitamin A treatment doses were found to reduce measle mortality by 65% and Afghanistan as a whole severely lacks necessary food aid [20,26]. This should be done while also targeting rural areas as they have the unique problem of being hard to reach with aid in general, thus it would be beneficial if agricultural training programs were conducted in rural areas to teach locals, support the economy, and provide them with the tools necessary to manage their businesses.

## 5 Conclusion

In conclusion, insufficient nutritional food supply, drastic rise in COVID-19 cases and financial instability in Afghanistan are one of the many factors that have contributed to the escalation of malnourished children in Afghanistan. Moreover, decrease in measle surveillance has increased mortality rate amongst undernourished children drastically due to their weak bodies. As a result, malnourished Afghan children are in dire need of financial and nutritional aid (specially vitamin A supplements) to help strengthen their immune systems against infectious disease like measles.

## Ethical approval

N/A.

## Sources of funding

None.

## Author contribution

Yumna Salman wrote the introduction, conclusion and organised references; Hira Anas Khan wrote the challenges and implications; Sean Kaisser Shaeen wrote the efforts and recommendation; Zarmina Islam conceived the idea and design, edited the revised draft; Mohammad Yasir Essar made the critical comments and revision. All authors revised and approved the final draft.

## Registration of research studies

Name of the registry: N/A.

Unique Identifying number or registration ID: N/A.

Hyperlink to your specific registration (must be publicly accessible and will be checked): N/A.

## Guarantor

N/A.

## Consent

N/A.

## Declaration of competing interest

None declared.
